# Interdaily Rest-Activity Variability and Risk of Dementia: The ARIC Neurocognitive Study

**DOI:** 10.1093/geroni/igaf122.1954

**Published:** 2025-12-31

**Authors:** Keran Chamberlin, Lacey Etzkorn, Anis Davoudi, Ryan Dougherty, Priya Palta, Amal Wanigatunga, Adam Spira, Jennifer Schrack

**Affiliations:** Johns Hopkins Bloomberg School of Public Health, Baltimore, Maryland, United States; Johns Hopkins Bloomberg School of Public Health, Baltimore, Maryland, United States; Johns Hopkins University, Baltimore, Maryland, United States; Rutgers University, New Brunswick, New Jersey, United States; UNC School of Medicine, Chapel Hill, North Carolina, United States; Johns Hopkins Bloomberg School of Public Health, Baltimore, Maryland, United States; Johns Hopkins Bloomberg School of Public Health, Baltimore, Maryland, United States; Johns Hopkins Bloomberg School of Public Health, Baltimore, Maryland, United States

## Abstract

Given mixed findings regarding the relationship between interdaily rest-activity variability and dementia risk, we hypothesize that interdaily variability of physical activity (PA) intensity and of rest-activity cycle each have distinct associations with dementia risk. We included 2332 dementia-free participants who had ≥3 days of ZioXT accelerometer data from the ARIC Neurocognitive Study Visit 6 (79±4.6 years, 58% female, 24% Black). Interdaily variability of PA intensity was operationalized as two measures: median absolute deviation (MAD) of total 24-hour activity and MAD of total activity during the most active 10 hours (M10). Interdaily variability of rest-activity cycle was defined in two ways: MAD of the start time of M10 and MAD of the start time of the least active 5 hours. Participants were followed until incident dementia, death, last contact, or December 31, 2022. After a median 5.2-year follow-up, 319 (13.7%) individuals developed dementia. Comparing the lowest (Q1) to the highest quartile (Q4) of MAD of total 24-hour activity, the cause-specific hazard ratio (HR) of dementia was 2.3 (95% confidence interval [CI]: 1.56, 3.46), with similar findings for MAD of total activity during M10. In contrast, the lower MAD of the start time of M10 was associated with lower risk of dementia (cause-specific HR for Q1 vs. Q4: 0.7 [95% CI: 0.51, 0.97]). The associations persisted after further adjusting for total 24-hour activity. Our results suggest that higher intensity PA (leading to higher interdaily variability) appears protective for dementia risk, while greater inconsistency of rest-activity cycle may induce greater risk.

